# Plaque Stabilization and Regression, from Mechanisms to Surveillance and Clinical Strategies

**DOI:** 10.31083/j.rcm2512459

**Published:** 2024-12-25

**Authors:** Xi Zhang, Huanhuan Feng, Yan Han, Xiaohang Yuan, Mengting Jiang, Wei Wang, Lei Gao

**Affiliations:** ^1^Senior Department of Cardiology, Sixth Medical Center of Chinese PLA General Hospital, 100853 Beijing, China; ^2^Medical School of Chinese PLA, 100853 Beijing, China; ^3^Emergency Department, First Medical Center of Chinese PLA General Hospital, 100853 Beijing, China

**Keywords:** atherosclerotic, plaque stabilization, plaque regression, lipid-lowing therapy, intravascular ultrasound, optical coherence tomography

## Abstract

With advances in therapies to reduce cardiovascular events and improvements in coronary imaging, an increasing number of clinical trials have demonstrated that treatments to reduce cardiovascular events in coronary artery disease are associated with favorable effects on atherosclerotic plaque size and characteristics. It has been observed that various drugs may induce plaque regression and enhance plaque stability after plaque formation. Numerous clinical trials have been conducted to verify the occurrence of plaque stabilization and regression and their beneficial effects on cardiovascular events. Using invasive imaging techniques such as intravascular ultrasound (IVUS) and optical coherence tomography (OCT), researchers have been able to gather evidence supporting the existence of coronary plaque stabilization and regression. In this review, we explore the possible mechanisms of plaque stabilization and regression, summarize the imaging features of plaque stabilization and regression, and assemble the evidence from clinical studies that have used different features as observational endpoints.

## 1. Introduction

Atherosclerosis is a chronic, progressive disease process with a long latency period, and clinical manifestations may not become apparent for two or three decades [1]. With increasing research into the pathogenesis of atherosclerosis, it is increasingly recognized that lipid deposition and macrophage infiltration at arterial wall lesions are reversible processes, and the hypothesis that the atherosclerotic process in humans can be reversed and regressed has persisted for decades [2]. After plaque formation, plaque volume and composition can be altered by a variety of therapies, including lipid lowering [3]. The use of invasive and non-invasive imaging techniques has made it possible to assess plaque burden and local plaque characteristics and to confirm that plaque stabilization and regression are real and reliable [4]. In recent years, study have confirmed the correlation between plaque stabilization and regression with reduced major adverse cardiovascular events (MACE) [5].

The aim of this review is to explore the possible mechanisms of plaque stabilization and regression, summarize the imaging features and clinical evidence, and predict the future research direction.

## 2. Methodology and Results

The researchers independently conducted a computerized literature search of three databases, PubMed, Embase, and Web of science, from 2018 to 2024. The search was conducted using the relevant search terms: “atherosclerotic plaque regression” or “plaque regression” and “plaque stabilization”, and more than 180 articles were retrieved. In addition, all the references of the retrieved articles were assessed for more information.

## 3. Definition of Plaque Regression

Plaque regression has traditionally been defined as an increase in lumen diameter as measured by coronary angiography [6]. Second, due to advances in intraplaque imaging, a reduction in atherosclerotic plaque volume as well as a reduction in markers related to plaque vulnerability can also be considered plaque regression [7, 8, 9]. However, there is no consensus about whether increased plaque stability is part of plaque regression.

## 4. Mechanism of Plaque Stabilization and Regression

Clinically, the development of therapies that result in regression and increased stability of atherosclerotic plaque is a desirable therapeutic goal for coronary heart disease, as most patients begin treatment after plaque formation. However, plaque regression is considered challenging due to the biological nature of advanced lesions, including necrotic cores, calcification, and fibrosis. Fortunately, many clinical studies have provided solid evidence of plaque stabilization and regression in humans following aggressive lipid-lowering therapy, giving researchers firm confidence to further explore the underlying mechanisms that regulate this complex process.

**Fig. 1.**
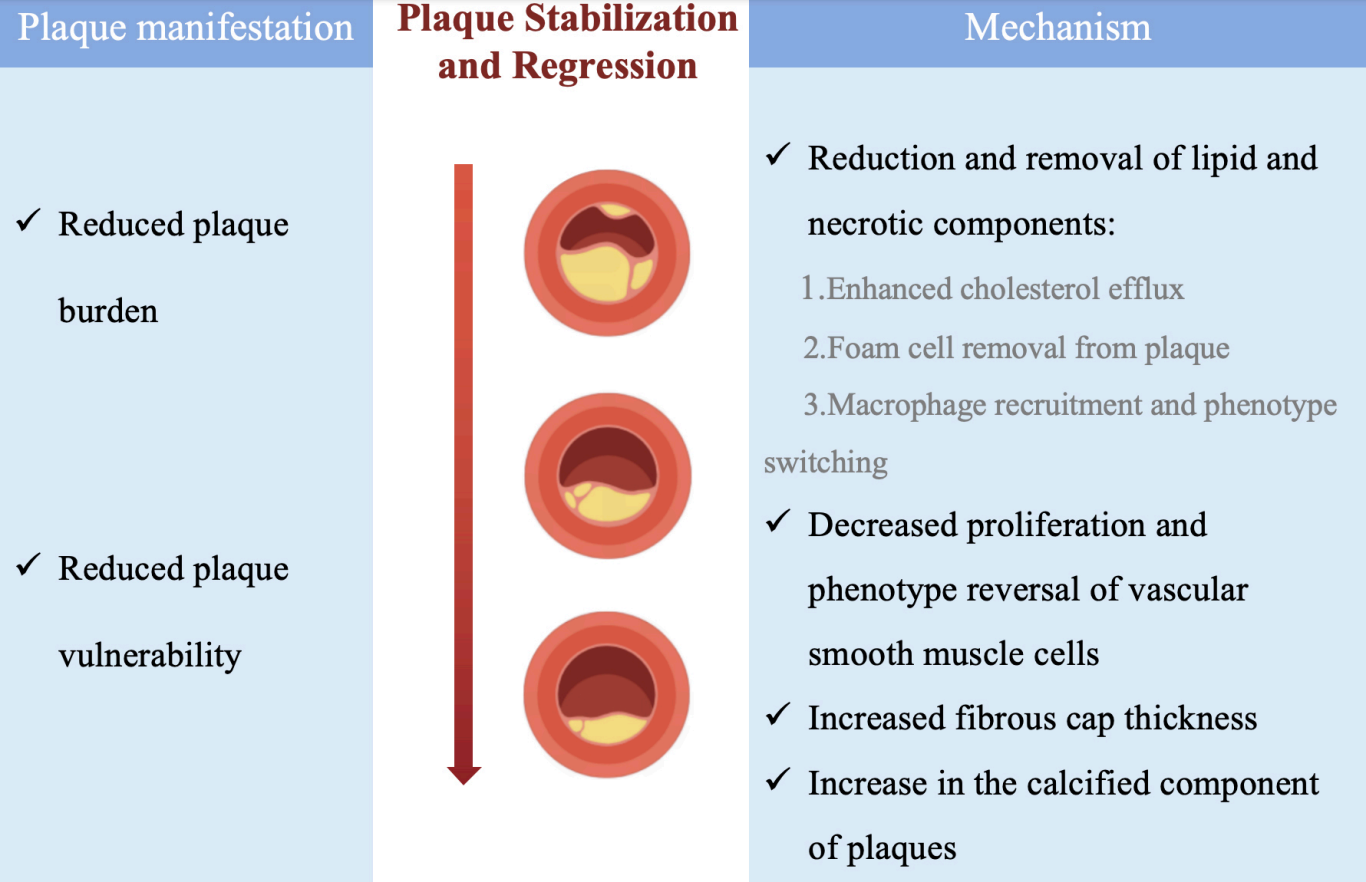
**Possible mechanisms of plaque stabilization and regression**. Manifestations and possible mechanisms of plaque stabilization and regression.

The mechanism of plaque stabilization and regression is not in itself equivalent to a reversal of the plaque development process, but rather consists of several important processes: the removal of lipids and necrotic components from the intima, the cessation of cell proliferation and undergoing phenotypic transformation, increase in the thickness of the fibrous cap, and increase in plaque calcification [10] (Fig. 1).

### 4.1 Reduction and Removal of Lipid and Necrotic Components 

The most important step in plaque regression is the removal of lipids and necrotic cores from the plaque by various routes.

#### 4.1.1 Enhanced Cholesterol Efflux

Animal study confirm that plaque regression is often accompanied by an increase in reverse cholesterol transport (RCT): the transfer of lipids from the periphery to the liver and their elimination from the body via the hepatobiliary pathway [11]. Promoting cholesterol efflux from macrophages is the first step in RCT. High-density lipoprotein (HDL) is essential to the RCT pathway as a cholesterol receptor. Several studies in large populations have shown that the ability of HDL to remove cholesterol is negatively correlated with cardiovascular disease [12, 13]. A preclinical evaluation showed that intravenous administration of autologous defatted HDL was able to result in a significant reduction in plaque volume by 6.9% [14].

One study silenced the expression of the macrophage surface protein Epsins, which enhances endocytosis of CD36 molecule to promote lipid uptake and impedes ATP-binding cassette subfamily G member 1 (ABCG1)-mediated cholesterol efflux, at the genetic level, and observed a reduction in necrotic core regions, a decrease in macrophage clusters, and a decrease in foam cell formation [15]. The results suggest that tilting the macrophage lipid balance toward cholesterol efflux may accelerate the achievement of plaque regression. In conclusion, enhanced transport of excess cholesterol from arteriolar lipid-rich lesions has become an important approach to anti-atherosclerotic drug development.

On the other hand, enhancing hepatic lipid uptake and clearance is also a feasible way to promote plaque regression. Ishigaki *et al*. [16] utilized viral transfection to ectopically express lectin-like oxLDL receptor-1 (LOX-1) in the liver to promote plasma oxidized low-density lipoprotein cholesterol (oxLDL-C) uptake and clearance. The results showed that the atherosclerotic plaques were almost completely regressed, strongly confirming the key role of lipid clearance in plaque regression.

#### 4.1.2 Foam Cell Removal from Plaque 

Apoptosis and necrosis of foamy macrophages form the necrotic core. There is growing evidence that removal of foamy macrophages is associated with increased plaque stability and can occur concurrently with a decrease in plaque volume [17].

Some immune cells are involved in foam cell clearance. A mouse model of atherosclerosis suggests that enrichment of plaque regulatory T cells (Treg) is a common feature of plaque volume reduction, and that Treg are required for reduction of plaque burden, reduction of inflammation, and repair of arterial wall tissues. RNA sequencing of immune cells in plaques and flow cytometry suggest that Treg in plaque regression may originate from splenic induction of generation. Second, to further validate whether Treg is required for plaque stabilization and regression, removal of Treg using a CD25 monoclonal antibody in a mouse model showed that reduction of Treg prevented plaque stabilization and regression achieved by lipid-lowering therapy, possibly by inhibiting macrophage migration, preventing macrophage phenotypic switching, and hindering macrophage pro-resolving functions [18]. In addition, peripherally derived CD4+ T cells can be involved in regulating the onset of plaque stabilization and regression by altering the balance of effector T cells and M1:M2 macrophages, promoting the clearance of dead cells to reduce plaque necrosis, and stimulating the production of lipid mediators for inflammatory regression.

In addition, an aortic graft model showed that the route of destination for foamy macrophages in plaques was the peripheral lymph nodes, and epigenomic analysis of the transcriptome of plaque foamy macrophages revealed that the Wnt-β-catenin pathway may be the specific mechanism by which macrophage motility is enhanced and migrates out of the plaque [19]. The macrophage retention factors Netrin1 and semaphorin 3E may be two potential mediators involved in the regulation of macrophage migration [20].

#### 4.1.3 Macrophage Recruitment and Phenotype Switching

However, macrophage recruitment is also thought to be responsible for plaque stabilization and regression, but this is not contradictory. Although macrophage recruitment and interactions with other cells directly induce plaque formation, it is also necessary for plaque stabilization and regression. Plaque stabilization and regression does not occur without newly recruited macrophages from the circulatory system.

A shift in macrophage polarization towards an M2 phenotype has also been suggested to be a feature of plaque regression. Rahman *et al*. [17] established an aortic graft model in which an enrichment of activated M2 macrophage markers was observed in plaque-incurring mice and confirmed that they were derived from newly recruited Ly6Chi monocytes by single-cell RNA sequencing. It was further found that it may induce M2 macrophage polarization by stimulating the peroxisome proliferator-activated receptor-γ pathway, which in turn promotes the clearance of apoptotic cells and debris through CD47 and tyrosine receptor-mediated cell proliferation [19]. In addition, more visual evidence comes from the treatment of atherosclerotic plaque mice with the M2 polarizing cytokine IL-13, where an increase in the M2 phenotype as well as plaque regression was observed. The signal transducer and activator of transcription 6 (STAT6) signaling pathway plays an important role in macrophage phenotypic transformation [21]. On the other hand, statin therapy, which reduces cardiovascular mortality, has been shown to exert a dual effect on plaque morphology, such as regression of atherosclerosis and increase of calcium deposition visible to the naked eye. The reason for this may be the ability of M2-type macrophages to induce osteoblast differentiation and smooth muscle cell maturation, thereby promoting calcium deposition, called macrocalcification. Clinically, the two types of plaque calcification have different implications, with macrocalcification leading to an increase in plaque stability, whereas microcalcification may be associated with plaque rupture [22].

In addition, study exploring the mechanisms of plaque regression has been conducted in humans. One study [23] achieved significant plaque regression by increasing the expression of proresolving lipid mediators in patients with stable angina pectoris (CAD) treated with statins, which was attributed to a decrease in the uptake of low-density lipoprotein cholesterol (LDL-C) by macrophages as well as enhanced phagocytosis by macrophages due to proresolving lipid mediators.

**Fig. 2.**
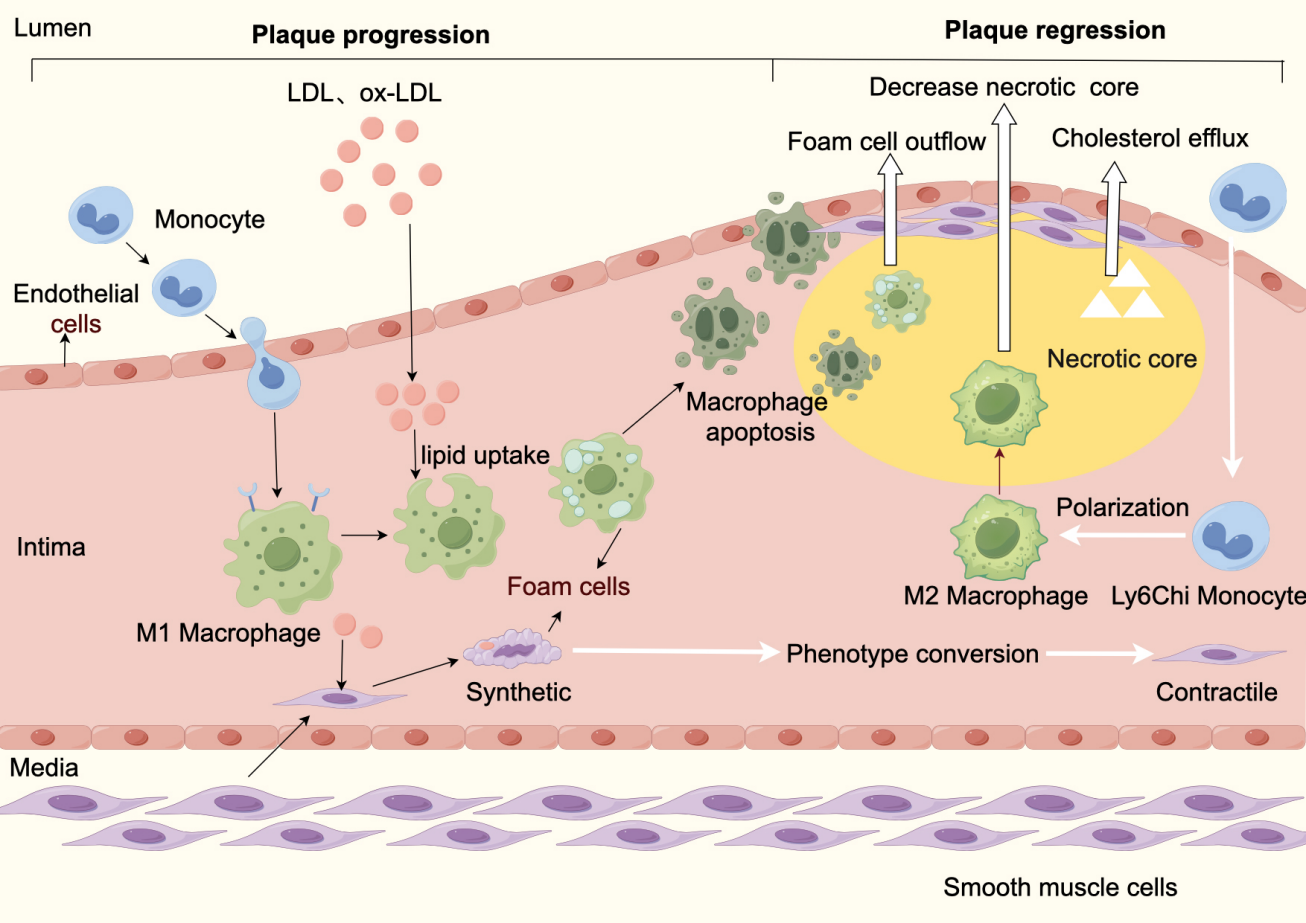
**Atherosclerosis progression and regression**. Plaque progression: Plaque formation begins with endothelial dysfunction caused by various pathogenic factors. Impaired endothelial function leads to increased permeability, which causes abnormal accumulation of lipid particles (mainly LDL) in the blood under the endothelium, which then attracts circulating monocytes to migrate to the sub endothelium and differentiate into macrophages, which phagocytose LDL-C to form foam cells. In addition, smooth muscle cells from the media shift from a contractile phenotype to synthetic phenotype phagocytizing lipids to form smooth muscle cell-derived foam cells. Foam cells apoptosis and secondary necrosis form a necrotic core, and synthetic-type smooth muscle cells proliferate and produce collagen, forming a fibrous cap and ultimately an atherosclerotic plaque. Plaque stabilization and regression: Lipid efflux and foam cell efflux cause a decrease in the necrotic core. LDL, low density lipoprotein cholesterol; Ox-LDL, oxidized low-density lipoprotein cholesterol; M1, pro-inflammatory macrophage phenotype; M2, anti-inflammatory macrophage phenotype.

In conclusion, promotion of reverse cholesterol transport, promotion of macrophage-derived foam cell plaque efflux, and promotion of M2-type macrophage transformation are feasible ways to enhance plaque lipid efflux and increase stability (Fig. 2).

### 4.2 Decreased Proliferation and Phenotype Reversal of Vascular Smooth Muscle Cells 

Smooth muscle cells can serve as another important source of foam cells by mediating cholesterol influx via surface receptors. In addition, certain pro-inflammatory cytokines and growth factors triggered by the inflammatory process can promote the migratory proliferative phenotype of smooth muscle cells, which can lead to remodeling of the overall structure of the arterial wall and promote plaque progression [24]. However, the role of smooth muscle cells in plaque regression is poorly understood. One study attempted to remove lipids from smooth muscle cells and inhibit their proliferation and observed plaque regression accompanied by phenotype transformation. Possible mechanisms are that macrophage migration from the plaque area mediates a reduction in the inflammatory process within the plaque and a reduction in the lipid component that promotes phenotype switching of pathologic smooth muscle cells [10].

At present, no studies have detected changes in the phenotype and number of smooth muscle cells in animal models, and it is not clear whether this is the mechanism of plaque regression.

### 4.3 Increase in the Thickness of the Fibrous Cap

Thin-cap fibroatheroma (TCFA) is a plaque morphology often considered to be prone to rupture and is usually defined as a plaque with necrotic nuclei and macrophages near the arterial lumen with a thin fibrous cap measuring <65 µm. The integrity of the fibrous cap, which may severely affect plaque stability, depends on collagen breakdown by interstitial collagen synthesized by smooth muscle cells and macrophages and by matrix metalloproteinases (MMPs) and other proteases produced mainly by macrophages. An increase in the thickness of the fibrous cap is seen as a reliable indicator of increased stability. The Food and Drug Administration (FDA) approved colchicine for the treatment of coronary atherosclerotic heart disease, and the LoDoCo2 trial [25] confirmed that colchicine intervention reduced the risk of primary composite cardiovascular events by 31%, which was previously thought to be attributable to anti-inflammatory effects. However, methotrexate did not produce the same effect, and the success of canamizumab inhibition has also been questioned. Given that both colchicine and canakinumab inhibited the interleukin-1β (IL-1β)-mediated pathway with different results, the researchers further found that the additional independent protective effect of colchicine came from converting the deleterious smooth muscle cell (SMC)-derived to protective myofibroblast-like cells which thickened, and thereby stabilized, the fibrous cap. It is well known that ACTA2+ myofibroblast-like cells are the main source of collagen within atherosclerotic plaque. This result explains the success of colchicine relative to other anti-inflammatory therapies and strongly suggests that targeting the transdifferentiation of plaque cells and thereby promoting an increase in fibrous cap thickness contributes to an improved prognosis for patients [25].

### 4.4 Increase in the Calcified Component of Plaques

Recent clinical studies have shown that statins can reduce plaque burden by decreasing the percentage of atherosclerotic plaque volume and total volume, while calcification volume increases [26]. Previous studies on the relationship between coronary calcification and plaque stability or instability have not been adequately investigated in clinical studies and remain controversial. However, recent pathologic and imaging study has shown that lamellar calcification is a marker of plaque stability, whereas minute, punctate, or fragmentary calcification is associated with early plaque or unstable plaque [26].

The ARCHITECT study characterized plaque throughout the coronary tree by coronary computed tomography angiography (CCTA) in patients treated with a combined lipid-lowering regimen and showed that the use of the combined lipid-lowering regimen was observed at follow-up to be associated with a significant reduction in plaque burden (–4.6%, *p* < 0.001), an increase in calcified plaque (+0.3%, *p* < 0.001), and a lower clinical event rate. The increase in calcified plaques can be linked and explained as one of the mechanisms by which plaque regression occurs [27].

Although imaging can assess metrics and estimate the incidence of plaque stabilization and regression, imaging tools do not capture the true complexity of the process, and the processes and mechanisms involved in the occurrence of plaque stabilization and regression remain to be discussed.

## 5. Imaging Characteristics of Plaque Stabilization and Regression

**Fig. 3.**
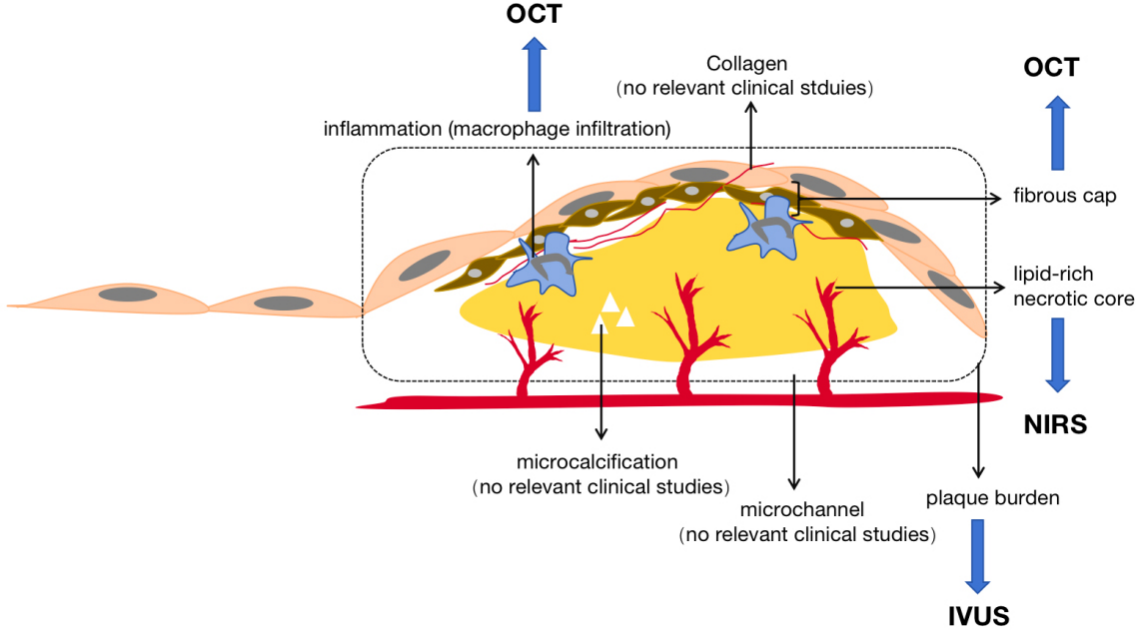
**Plaque imaging modalities**. Plaque imaging modalities. IVUS, intravascular ultrasound, used for plaque burden measurement; OCT, optical coherence tomography, used for measurement of plaque vulnerability characteristics; NIRS, near infrared spectroscopy, used for measurements on lipid-rich cores.

Analyzing the definition of plaque regression, the change in plaque volume is the most intuitive indicator of the occurrence of plaque regression. In addition, significant changes in plaque characteristics are also considered diagnostic criteria for increased plaque stability [28]. Multiple imaging strategies are available for comprehensive assessment of plaque. They can be categorized as invasive and noninvasive. Invasive imaging modalities include conventional coronary angiography, intravascular ultrasound (IVUS), optical coherence tomography (OCT), near-infrared spectroscopy (NIRS), and other intravascular imaging. Non-invasive imaging modalities include computed tomography angiography (CTA) and cardiac magnetic resonance imaging (MRI) (Fig. 3).

### 5.1 Invasive Modalities 

Conventional coronary angiography (CAG) provides information about the vessel lumen and can identify segments of vessels that visually cause significant narrowing of the lumen. However, because it is limited to the lumen and lacks information about plaque burden and composition, it is less commonly used to assess plaque stabilization and regression.

IVUS is the traditional standard for assessing plaque burden in clinical trials and has been used as a diagnostic imaging modality for plaque stabilization and regression imaging in many clinical studies. IVUS not only distinguishes plaques into fibrous, fibrofatty, calcified, and calcified necrotic subtypes, but also accurately measures plaque burden including atherosclerotic plaque area, total atherosclerotic plaque volume (TAV), and percent atherosclerotic plaque volume (PAV) [29]. These parameters can be measured over time to quantify plaque progression and regression [30].

OCT, a light-based modality for intravascular imaging of catheter delivery, has the advantage of high-resolution characterization of the surface elements of the vessel wall, allowing assessment of plaque stability by differentiating plaque morphology, estimating fibrous cap thickness, and showing the extent of macrophage infiltration. In recent years, many clinical trials have used OCT as an imaging modality to assess the effects of various drugs on plaque regression using plaque characteristics and composition as observational endpoints (including lipid content, presence of macrophage clusters, microcalcifications), with positive results. OCT is the gold standard for correctly measuring fibrous cap thickness *in vivo*, and there is a good correlation between the examined fibrous cap thickness and histology [31]. TCFA characterized by large necrotic cores and a thin fibrous cap overlying them are considered precursor lesions to plaque rupture leading to acute events. Radiofrequency (RF)-IVUS and OCT are the two intracoronary imaging techniques for detecting TCFA. Diagnosis of RF-TCFA is visually assessed based on the number and location of necrotic cores, regardless of cap thickness. Previous clinical studies have demonstrated that the presence of TCFA defined by RF-IVUS-defined presence of TCFA is associated with future MACE. A direct comparison regarding the diagnostic assessment of TCFA between RF-IVUS and OCT was confirmed by the IBIS-4 study [31] to be significantly inconsistent. This discrepancy may be due to the resolution of IVUS and the inherent limitations of assessing the necrotic core [31]. However, due to its poor penetration, it is often not used for plaque burden measurement [3]. The presence of macrophage clusters is one of the OCT predictors of future coronary events. The ability of plaque OCT to recognize macrophages has been validated, however different subtypes of macrophages in the plaque background could not be distinguished [32]. NIRS allows for quantitative analysis of lipid content in plaques and is used to assess the effect of lipid-modifying therapies on lipid content in plaques. The LRP and the PROSPECT II study reported that the lipid core burden index assessed by NIRS was significantly associated with future MACE [31]. While NIRS is the gold standard for *in vivo* lipid identification, it cannot distinguish between superficial and deep lipid pool. Thus, it is usually combined to IVUS catheter or more recently to OCT. Undeniably, the combination of NIRS and IVUS is still unable to accurately determine the location of plaque lipid as the lipid signal is characterized by a circumferential arc of its location around the IVUS image although not addressing the depth of the signal.

### 5.2 Non-Invasive Modalities 

With advancements in technology, the current trend favors the use of noninvasive imaging for plaque assessment that can provide additional information beyond stenosis and plaque with minimal risk. Previous study has shown that plaque measurements using cardiac CTA are highly accurate compared to IVUS [33]. CTA has been shown to identify high-risk plaques associated with plaque vulnerability features. It can also show a strong correlation with macrophage infiltration observed on OCT by positive remodeling and low attenuation of plaque in the image [34]. Recent consensus has discussed the advantages and disadvantages of various imaging modalities for plaque and stenosis assessment, and CTA is considered the most suitable imaging modality for plaque assessment, making it the most valuable technique for practical clinical situations. In contrast, IVUS was considered suitable for assessing plaque composition and detecting culprit plaques [35].

Cardiac MRI has great advantages in assessing coronary artery wall thickness and positive remodeling, but it is not commonly used for coronary artery assessment because of the high level of expertise required for image recognition, the long examination time, and its applicability to large arteries [36].

## 6. Clinical Strategies 

Current clinical interventions for plaque stabilization and regression include pharmacologic as well as nonpharmacologic therapies. Pharmacological therapies are divided into two categories based on whether they target LDL-C.

### 6.1 Clinical Evidence for LDL-C as a Target for Intervention

Lowering LDL-C levels is the mainstay of plaque stabilization and regression. Most current clinical trials have focused on whether lipid-lowering therapies induce plaque regression or increase plaque stability (Table 1, Ref. [37, 38, 39, 40, 41, 42, 43, 44, 45, 46, 47, 48, 49, 50]). High-quality clinical evidence exists to support the use of various lipid-lowering regimens to stabilize and regress coronary plaque.

**Table 1.** **Clinical study on the evaluation of lipid-lowering drugs on atherosclerotic plaque stabilization and regression**.

First author, year	Treatment	Imaging mode	Sample size	Follow-up weeks	Positive indicators related to plaque regression	Main findings
Nicholls, 2011 [38]	Atorvastatin 80 mg/d	IVUS	1039	104 weeks	PAV	The high-intensity lipid-lowering treatment group had lower LDL-C levels, a greater reduction in PAV (–1.22% vs. 0.99%), and a more pronounced effect on TAV (–6.39 mm^3^ vs. –4.42 mm^3^).
	Rosuvastatin 40 mg/d					
Okazaki, 2004 [37]	Atorvastatin 20 mg/d	V-IVUS	70	6 months	PAV	Plaque volume was significantly reduced in the atorvastatin group (13.1 ± 12.8% decrease) compared with the control group (8.7 ± 14.9% increase, *p * < 0.0001).
						Percent change in plaque volume showed a significant positive correlation with follow-up LDL-C level (R = 0.456, *p * < 0.0011) and percent LDL-C reduction (R = 0.612, *p * < 0.0001).
Nissen, 2006 [49]	Rosuvastatin 40 mg/d	IVUS	507	24 months	PAV, TAV	The mean (SD) change in PAV for the entire vessel was 0.98% (3.15%); Change in TAV showed a 6.8% median reduction.
Nissen, 2005 [50]	Atorvastatin 80 mg/d	IVUS	502	18 months	PAV, TAV	Progression of coronary atherosclerosis occurred in the pravastatin group (2.7%; 95% CI: 0.2% to 4.7%; *p* = 0.001) compared with baseline. Progression did not occur in the atorvastatin group (−0.4%; 95% CI: −2.4% to 1.5%; *p* = 0.98) compared with baseline.
	Pravastatin 40 mg/d					
Tsujita, 2015 [39]	Atorvastatin 10 mg/d	IVUS	202	9–12 months	PAV	The absolute change in PAV did show superiority for the dual lipid-lowering strategy (–1.4%; 95% CI: –3.4% to –0.1% vs. –0.3%; 95% CI: –1.9% to 0.9%, *p* = 0.001).
	Atorvastatin + Ezetimibe 10 mg/d					
Nicholls, 2016 [40]	Evolocumab 420 mg/L month	IVUS	968	76 weeks	PAV, TAV	The primary efficacy parameter, PAV, increased 0.05% with placebo and decreased 0.95% with evolocumab (difference, −1.0% [95% CI: −1.8% to −0.64%], *p * < 0.001). The secondary efficacy parameter, normalized TAV, decreased 0.9 mm^3^ with placebo and 5.8 mm^3^ with evolocumab (difference, −4.9 mm^3^ [95% CI: −7.3 to −2.5], *p * < 0.001).
Yano, 2020 [42]	Rosuvastatin 5 mg/d	OCT	58	4–12 weeks	Lipid content, Macrophage content	OCT analysis revealed that the increase in fibrous-cap thickness and decrease in macrophage grade were greater with a narrower lipid arc and shorter lipid length.
	Rosuvastatin 5 mg/d + Evolocumab 140 mg/2 weeks					
Sugizaki, 2020 [48]	Alirocumab 75 mg/2 weeks + Rosuvastatin 10 mg/dL	OCT	24	36 weeks	Lipid content, Macrophage grade	Both the absolute increase (primary endpoint) and percentage increase in FCT were significantly greater in the alirocumab group than in the standard-of-care group (absolute change: 140 mm [78 to 163 mm] vs. 45 mm [10 to 78 mm] [*p* = 0.002]; percentage change: 273% [155% to 293%] vs. 100% [20% to 155%] [*p* = 0.004]).
	Rosuvastatin 10 mg/dL					The macrophage grade decreased significantly in the alirocumab group but not in the standard-of-care group, and the percentage change in macrophage grade was significantly greater in the alirocumab group (–28.4% [–35.3% to –19.0%] vs. –10.2% [–25.3% to 4.3%], *p* = 0.033).
Ota, 2022 [43]	Evolocumab 140 mg/2 weeks	NIRS-IVUS	53	12 months	TAV, PAV, Lipid content	The percent reduction in normalized atheroma volume and absolute reduction in percent atheroma volume (PAV) were also significantly greater in the PCSK9i group (*p * < 0.001 for both).
						Furthermore, the PCSK9i group showed greater regression of maximal lipid core burden index for each of the 4-mm segments (maxLCBI4mm) than the control group (57.0 vs. 25.5, *p* = 0.010).
Hattori, 2012 [44]	Pitavastatin 4 mg	OCT	42	9 months	FCT	Fibrous cap thickness over time between the pitavastatin and diet groups were highly significant.
Habara, 2014 [46]	Ezetimibe (10 mg/day) + Fluvastatin (30 mg/day)	OCT	63	9 months	FCT	The change in the fibrous cap thickness was significantly greater in the ezetimibe + fluvastatin group (0.08 ± 0.08 mm vs. 0.04 ± 0.06 mm, *p * < 0.001).
	Fluvastatin (30 mg/day)					
Nicholls, 2022 [45]	Evolocumab 400 mg/L month	OCT	161	52 weeks	FCT, PAV, Lipid content	The evolocumab group demonstrated a greater increase in minimum fibrous cap thickness (+42.7 vs. +21.5 mm, *p* = 0.015) and decrease in maximum lipid arc (–57.5° vs. –31.4°, *p* = 0.04) and macrophage index (–3.17 vs. –1.45 mm, *p* = 0.04) throughout the arterial segment.
Räber, 2022 [47]	Alirocumab 75 mg/2 weeks	IVUS, OCT, NIRS	300	52 weeks	FCT, Lipid content	At 52 weeks, mean change in percent atheroma volume was −2.13% with alirocumab vs. −0.92% with placebo (difference, −1.21% [95% CI: −1.78% to −0.65%], *p * < 0.001).
						Mean change in maximum lipid core burden index within 4 mm was −79.42 with alirocumab vs. −37.60 with placebo (difference, −41.24 [95% CI: −70.71 to −11.77], *p* = 0.006).
						Mean change in minimal fibrous cap thickness was 62.67 µm with alirocumab vs. 33.19 µm with placebo (difference, 29.65 µm [95% CI, 11.75 to 47.55], *p* = 0.001).
Oh, 2021 [41]	Atorvastatin 10 mg + Ezetimibe 10 mg	NIRS-IVUS	41	12 months	PAV, Lipid content	The combination of atorvastatin 10 mg and ezetimibe 10 mg showed comparable LDL-C lowering and regression of coronary atherosclerosis in the intermediate lesions, compared with atorvastatin 40 mg alone.
	Atorvastatin 10 mg					

IVUS, intravascular ultrasound; V-IVUS, virtual intravascular ultrasound; OCT, optical coherence tomography; NIRS, near infrared spectroscopy; PAV, percentage of atherosclerotic plaque volume; TAV, total atherosclerotic plaque volume; FCT, fiber cap thickness; LDL-C, low-density lipoprotein cholesterol; PCSK9i, proprotein convertase subtilisin/kexin type 9 inhibitor.

Most of the current clinical studies on lipid-lowering therapy to induce plaque regression and promote plaque stabilization have used different imaging modalities, defined according to different plaque components and features. Overall, the observational metrics are categorized into the following 4 types.

#### 6.1.1 Reduction in Percent Atheroma Volume (PAV) and Total Atheroma Volume (TAV)

TAV appears to be the logical definition of plaque regression [51]. Therefore, many clinical trials have used both PAV and TAV to reflect changes in plaque volume and to define whether plaque regression is occurring.

The idea that statins can significantly reduce plaque volume by lowering LDL-C has been recognized by researchers. Study has further confirmed the positive correlation between LDL-C reduction and plaque volume reduction [37]. Increasing the dose of statins is a means of achieving lower LDL-C levels. A study confirmed lower LDL-C levels in the high-intensity lipid-lowering therapy group, with a greater reduction in PAV (–1.22% vs. 0.99%). The effect on TAV was even more pronounced (–6.39 mm^3^ vs. –4.42 mm^3^) [38]. Although PAV and TAV are well-validated measures of IVUS-derived plaque volume, PAV incorporates the amount of plaque present in relation to the adaptive response of the vessel wall. As such, the concomitant arterial remodeling response of the vessel wall affects the calculation of PAV. PAV has thus become the primary efficacy endpoint for most trials using serial IVUS to assess changes in coronary atheromatous plaque volume [52]. More interestingly, in an analysis of clinical factors associated with changes in coronary artery volume after statin therapy, the researchers found that female patients on the same regimen were more likely to experience plaque regression and greater differences in PAV than male patients, and multivariate analyses also showed that women acted as independent predictors of PAV regression [52].

According to the latest global guidelines, the target LDL-C level for patients at very high cardiovascular risk is <55 or <70 mg/dL, and statins have a limited ability to lower LDL, and many patients do not achieve optimal LDL-C lowering with upfront statin therapy alone [53]. For patients whose LDL-C levels remain suboptimal on statins and who cannot tolerate high-intensity statins, a combination of other types of lipid-lowering drugs, such as the cholesterol uptake inhibitor ezetimibe and a PCSK9 inhibitor, has become a basic strategy for plaque reversal therapy. What’s more, the PRECISE-IVUS [39] study showed superior changes in plaque volume PAV when statins were combined with other types of lipid-lowering drugs (–1.4% vs. –0.3%). The proportion of patients experiencing plaque regression was also significantly higher (78% vs. 58%). The GLAGOV [40] study showed satisfactory results that statins combined with PCSK9i significantly reduced TAV (–5.8 mm^2^ vs. –0.9 mm^2^) and PAV (–0.95% vs. +0.05%) and induced more plaque regression (64.3% vs. 47.3%). Increasing LDL-C clearance and exerting an anti-inflammatory effect may be the probable reason why proprotein convertase subtilisin/kexin type 9 inhibitor (PCSK9i) induces plaque regression. In conclusion, PCSK9i was able to dose-dependently reduce LDL cholesterol by approximately 60% in addition to statin therapy and further reduce the risk of cardiovascular events. And can be a good alternative for patients who cannot tolerate high-intensity statins [41]. Inclisiran, a PCSK9 siRNA that has been launched in China in 2023, has comparable lipid-lowering ability to PCSK9 inhibitors and lasts longer [54]. The current study on Inclisiran has demonstrated the long-term efficacy and safety of its additional LDL lowering with clinical cardiovascular outcomes as the primary endpoint. One of these studies, ORION-10, also demonstrated that Inclisiran was associated with a reduction in ApoB and an increase in HDL [55]. However, overall, its use is still in phase III clinical trials and studies on whether it correlates with the occurrence of plaque regression are lacking. In conclusion, the effect of statins on plaque regression depends not only by the absolute value of LDL, but it also varies according to the difference from the baseline to follow-up values as demonstrated by the study IBIS-4 [56]. The current acute coronary syndromes (ACS) guidelines recommend not only to reduce LDL value below a specific threshold, but also to reduce it of more than 50% [57].

The ability of LDL-C lowering to induce atherosclerotic plaque regression has been recognized by researchers. However, the relationship between lipid therapy-induced plaque regression and the occurrence of MACE remains controversial. Researchers now believe that plaque regression achieved by therapeutic regimens targeting LDL-C reduction does correlate with a favorable prognosis. Previous studies have confirmed that intensive lipid-lowering regimens can control lipids below recommended levels and reduce the incidence of MACE by 2.2% in absolute terms and 22% in relative terms [58, 59]. Similarly, a meta-analysis of 17 lipid therapy trials showed that a 1% reduction in mean PAV induced by treatment of dyslipidemia was associated with a 20% reduction in the incidence of MACE [5]. A systematic review and meta-analysis published in the journal of the American Medical Association (JAMA) in 2020 also used PAV as a surrogate for plaque regression, pooled 23 studies related to lipid-lowering therapy, and concluded that 1% plaque regression was associated with a 14%–25% reduction in the incidence of MACE, providing strong support for changes in PAV as a surrogate marker for MACE [56]. However, we should not oversimplify the correlation between PAV and MACE. Future studies will need to demonstrate this causal relationship by temporally sequencing changes in PAV and MACE events in individuals. What remains constant, however, is that lowering LDL cholesterol levels always seems to benefit patients who already have atherosclerotic disease.

However, imaging studies using serial IVUS have demonstrated only modest reductions in atherosclerotic plaque burden despite low (<70 mg/dL) [38] or very low (<40 mg/dL) [40] LDL-C levels during lipid-lowering therapy. The discrepancy between the significant increase in clinical benefit and the moderate plaque burden change as defined by IVUS suggests that the effects produced by intensive lipid-lowering therapy on plaque composition and the increase in plaque stability are more dominant. For example, reduction of necrotic cores in plaques, plaque calcification, increased fibrous cap thickness, and decreased macrophage infiltration.

#### 6.1.2 Reduction of Lipid-Rich Necrotic Core

Radiofrequency IVUS analysis has shown that statins induce favorable changes in plaque composition, leading to a decrease in lipid body mass and an increase in fiber content, and are more effective in reducing lipid composition when combined with PCSK9i. For example, the Yano study [42] from 2020 used OCT as an imaging method and observed a significant reduction in lipid length and maximal lipid arc in the lipid-lowering treatment group (–40° vs. –24°). In addition, the Ota *et al*. [43] used NIRS-IVUS as an imaging tool for semi-quantitative detection of plaque lipids and similarly observed that a lipid-lowering regimen significantly reduced lipid composition, decreased formation of lipid necrotic cores, and enhanced plaque stability.

#### 6.1.3 Plaque Calcification

Some researchers believe that statins may contribute to plaque stability by making these macrocalcifications more integrated and denser [60]. The SATURN study [61] performed serial IVUS measurements in patients treated with statins and showed that plaque regression was accompanied by an increase in dense calcium volume with no change in fiber or necrotic core tissue volume. The first trial using CTA to assess plaque in 2013 showed that statins significantly reduced low-attenuation and noncalcified plaque, which is one of the criteria for high-risk plaque [62].

Calcified plaques are considered the most stable form of plaque [63]. Paradoxically, some investigators have also suggested that plaque calcification is characteristic of progressive plaques and that the pattern and distribution of calcification correlates with the severity of CAD, with microcalcification more likely to be associated with vulnerable plaques with clinical events [64]. Therefore, the potential mechanisms and types of statin-mediated calcification deserve further investigation.

#### 6.1.4 Increase in Fiber Cap Thickness

Fibrous cap thickness as assessed by OCT was an important determinant of plaque vulnerability. Increased fiber cap thickness is a pathologic manifestation of increased plaque stability. Statin therapy after acute myocardial infarction has been reported to cause an increase in fibrous cap thickness [65]. Kousuke *et al*. [44] performed prospective OCT in patients with stable angina pectoris and showed a significant increase in fibrous cap thickness after statin treatment compared to baseline (140 µm vs. 189 µm), which was not significant in the control group. In addition, serial OCT analyses in patients with stable angina have been reported to show that the percentage reduction in LDL-C with statin therapy correlates with the percentage increase in fibrous cap thickness [45]. A clinical study conducted by Habara *et al*. [46] used OCT as an imaging modality to analyze fibrous cap thickness, a metric associated with plaque vulnerability, and the results suggested that the statin combined with ezetimibe group had a more pronounced increase in fibrous cap thickness (0.08 mm vs. 0.04 mm), a significant reduction in lipid plaque angle, and significantly better plaque regression. In addition, combination with PCSK9i also increased fibrous cap thickness in lipid-rich plaques compared with statin monotherapy, as confirmed by the HUYGENS [45] study.

From the perspective of imaging methods to observe plaque characteristics, some researchers believe that OCT is not the best way to observe atherosclerotic plaques with thin fibrous caps and may lead to false-positive results due to the presence of microcalcifications, foam cells, and thrombi [66]. Therefore, researchers hope to improve the resolution and sensitivity of plaque characterization using multimodal imaging. The PACMAN-AMI study [47] was the first to evaluate the effect of high-intensity statins in combination with PCSK9 inhibitors on plaque using simultaneous IVUS, OCT, and NIRS imaging and showed that the combination therapy resulted in satisfactory plaque regression. Surprisingly, the study also confirmed the benefits of PCSK9 inhibitors in improving plaque stability even with the use of high-intensity statins. Therefore, current guidelines recommend the use of PCSK9 inhibitors in combination with statins and ezetimibe in patients at very high risk of atherosclerotic cardiovascular disease when maximum tolerability of statins and ezetimibe remains unsatisfactory [67].

#### 6.1.5 Reduction of Macrophage Infiltration

Decreased macrophage content may also serve as a surrogate marker for increased plaque stability [68]. The 2020 ALTAIR study [48] also observed a significant reduction in macrophage grade and a greater percentage change in the statin plus PCSK9i group (–28.4% vs. 10.2%). The likely reason for this is the lower LDL-C levels induced by the combination therapy. The results provide possible mechanistic insights into the efficacy of adding alirocumab to standard-dose statins to improve clinical outcomes.

### 6.2 Clinical Evidence for Non-LDL-C as a Target for Intervention

However, despite the substantial reduction in LDL cholesterol because of lipid-lowering therapy, residual cardiovascular risk may remain. Mean on-treatment LDL cholesterol levels in SATURN [61] were the lowest achieved in any previously conducted atherosclerosis imaging study (62 mg/dL in patients taking Rosuvastatin and 70 mg/dL in patients taking Atorvastatin), but plaque progression was still present in one-third of patients. This suggests that alternative therapeutic strategies should still be actively sought, to reduce the burden of atherosclerosis.

Increasing blood HDL levels is also a hot topic of research in plaque stabilization and regression [69]. Steven *et al*. [70] investigated the role of elevated HDL on plaque volume and observed that patients given different doses of complex HDL had a mean decrease in plaque volume of 4.2% after 5 weeks of treatment and that HDL levels were positively correlated with plaque regression. However, there is also study in which plaque regression was not observed with HDL-mimicking drugs, so the ability of elevated HDL to promote plaque regression needs to be confirmed in further large randomized controlled trials [29].

In addition, residual inflammatory risk may be an important cause of cardiovascular events beyond cholesterol [19]. Schuett *et al*. [71] observed significant plaque regression in mice using inhibitors of IL-6 signaling. However, there are fewer clinical trials examining changes in plaque composition and volume after anti-inflammatory therapy. The exact relationship between anti-inflammatory therapy and plaque regression deserves further exploration.

In addition to inflammation, the risk of cardiovascular disease that remains after well-controlled LDL levels may be due to elevated triglyceride (TG)-rich lipoproteins, which are common dyslipidemias in patients with diabetes and metabolic diseases. A large, randomized trial using icosapent ethyl (IPE) showed that lowering triglycerides resulted in a significant reduction in adverse cardiovascular events in statin-treated patients and was proportional to the blood concentration of eicosapentaenoic acid (EPA) [72]. However, EPA/docosahexaenoic acid (DHA) blends, which possess similar triglyceride-lowering effects, did not show the same benefits in the trial. The inconsistency of the results has prompted further investigation into the possible mechanisms. Researchers believe that EPA in combination with statins maintains normal membrane cholesterol distribution, enhances endothelial function, and improves features associated with plaque stability. In addition, researchers believe that the apparent benefits of IPE in multiple trials may stem from multiple effects associated with therapeutic levels of EPA, not just triglyceride lowering. These effects include alterations in platelet function, inflammation, cholesterol distribution, and endothelial dysfunction [72].

### 6.3 Other Aspects

In addition to medication, lifestyle changes to control other diseases can affect the degree of plaque regression. Examples include quitting smoking, controlling weight, avoiding comorbid diabetes, lowering blood pressure, and increasing exercise.

It is well acknowledged that atherosclerosis is a major cardiovascular complication of diseases associated with chronic inflammatory status, increased oxidative stress and disorders of lipid metabolism. Systemic oxidative stress states predispose LDL to oxidation, forming oxidized LDL, which displays pro-atherosclerotic activity through a complex mechanism. Oxidized LDL cholesterol-rich oxidation products, also known as oxysterols, can exert various biological effects on vascular cells such as promoting apoptosis, inducing oxidative stress and cytotoxicity involved in plaque formation and destabilization, which have been well documented. It is now well demonstrated that oxidative stress is a determinant for the formation of oxysterols as well as signaling pathways evoked by deleterious oxysterols [73]. Therefore, the protective effect of reducing the production of oxysterols through antioxidant therapy is valuable and deserves further investigation. Cigarette smoking, a major health hazard, contributes to atherosclerosis, thrombosis, and inflammation through multiple mechanisms, including endothelial dysfunction and increased oxidative stress. Oxygen radical-mediated oxidative stress plays a central mechanism in smoking-mediated atherosclerotic disease. These free radicals may come directly from cigarette smoke or indirectly from endogenous substances [74]. And increased oxidative stress is largely associated with prothrombotic effects (increased platelet reactivity, decreased endogenous fibrinolysis, and lipid peroxidation) and inflammatory responses to the vessel wall. Furthermore, antioxidants or drugs that reduce oxidative stress have been shown to ameliorate or reverse the prothrombotic and proinflammatory features associated with smoking [74].

In a very small, randomized study published in 1990, 28 patients with coronary atherosclerosis were randomly assigned to receive intensive lifestyle changes, including smoking cessation, or usual care. After 1 year, percent diameter coronary stenosis assessed by coronary angiography was reduced from a mean of 40.0% to 37.8% in the intensive life-style group and increased from a mean of 42.7% to 46.1% in the control group [75]. The findings tentatively suggest that smoking cessation has an effect on volume regression after coronary plaque formation. In addition, much of the literature strongly suggests that smoking adversely affects all stages of atherosclerosis, for example, by promoting thrombosis or activating MMPs, which promote the formation and rupture of vulnerable plaques, thereby triggering acute events [76]. Zhang *et al*. [77] divided patients with acute coronary syndromes (ACS) after percutaneous coronary intervention (PCI) into smoking cessation group, persistent smoking group, and nonsmoking group. All three groups were treated with statins after surgery, and OCT was used to focus on the morphology of non-culprit plaques. It was found that the persistent smoking group had a smaller fibrous cap thickness and a higher incidence of TCFA compared with the other two groups. It was concluded that persistent smoking attenuated the effect of statin therapy on plaque stabilization in ACS patients. The results of this study suggest that smoking cessation may have a stabilizing effect on plaques by increasing fibrous cap thickness and improving plaque morphology [77].

Despite the paucity of data on plaque stabilization and regression after smoking cessation, it is undeniable that smoking is an important risk factor for patients with thrombotic coronary events (especially plaque erosion), and smoking cessation significantly reduces acute events by decreasing coronary thrombosis and attenuating the inflammatory response [78].

Previous study has found that the higher the patient’s body mass index, the greater the offset of plaque regression [79]. In addition, diabetes mellitus has a negative impact on plaque regression. The TRUTH study [80] confirmed that diabetes mellitus significantly impairs the plaque stabilizing effects of statins through complex mechanisms, including activation of hematopoiesis, increased inflammatory cell infiltration, and impeded macrophage polarization. On the other hand, the PESA study confirmed that factors such as non-smoking and being female may promote the occurrence of plaque regression [81]. In addition, the study has confirmed the positive effect of exercise intensity on plaque regression [82]. However, because the available data are low in quality, whether lifestyle modification has clinically significant effects on coronary plaque stabilization and regression remains uncertain.

In conclusion, pharmacologic therapy is the cornerstone of reducing plaque volume and enhancing plaque stability, on top of which we should also actively control other factors that affect the plaque volume and stability in order to minimize the occurrence of clinical events.

## 7. Future Directions

Favorable results from clinical studies in recent years have underscored the importance of achieving very low LDL-C levels in patients at high cardiovascular risk and have emphasized that combining multiple types of lipid-lowering agents provides clinical benefit in achieving these effects in most patients. Combination lipid-lowering therapy has become a common strategy to achieve plaque regression through greater reductions in LDL-C levels. However, it has not been established whether combination therapy with ezetimibe and statins is more effective than statins when LDL cholesterol levels are comparable. Although available data suggest that the lower the LDL cholesterol level, the greater the degree of coronary plaque regression. However, the long-term safety of very low LDL cholesterol levels remains to be investigated and confirmed by clinical trials.

Through the initial elucidation of the mechanisms underlying the onset of plaque regression and plaque stabilization, increasing attention has been paid to the role of inflammatory cells and inflammatory factors in this context, and more basic studies targeting the reduction of inflammation levels and the promotion of cellular phenotypic shifts may be needed in the future to confirm the onset of plaque regression and enhanced plaque stability. In addition, the role of neovascularization in promoting plaque formation and progression has been emphasized by researchers. The local hypoxic environment of plaques can upregulate proangiogenic factors to trigger neovascularization. Pathological, morphological, and functional characteristics of neovascularization such as morphologic disturbances, loss of basement membrane and pericytes, and abnormal increase in permeability allow for the delivery of inflammatory cells and lipoproteins to the lesion site, thereby exacerbating the lipid and inflammatory microenvironment within the plaque and promoting plaque formation and destabilization [83]. Animal study has demonstrated that inhibition of pathologic neovascularization facilitates increased plaque stability, as evidenced by a decrease in lipid content and macrophage accumulation. The likely mechanism is that protocatechuic aldehyde increases pericyte proliferation, migration, and adhesion, which serves to increase pericyte coverage of plaques and reduce vascular endothelial growth factor-A production, inhibiting plaque neovascularization. In addition, it can alleviate oxidized LDL-induced pericyte dysfunction and maintain capillary structure and stability [84].

Furthermore, the occurrence of progression of different plaque phenotypes and plaque regression has not been studied. It has been suggested that lipid-rich plaques have the most therapeutic value with the possibility of reversal with intensive LDL-lowering therapy and control of risk factors, whereas calcified plaques, even when LDL is lowered to very low levels, are less likely to be reversible in such plaques. What is certain, however, is that the plaque phenotype may change over time as a result of drug use and episodes of subclinical events [32].

## 8. Conclusions

There is increasing clinical evidence that patients with coronary atherosclerosis benefit from lipid-lowering therapy and that plaque stabilization and regression improve patient survival. However, various imaging techniques remain a surrogate endpoint, and plaque volume reduction and composition changes should not be interpreted as equivalent to clinical benefit in the prevention of cardiovascular events [38]. Despite these limitations, we believe that the effectiveness of currently used drugs in reducing plaque volume and increasing plaque stability through certain mechanisms is noteworthy. In addition, although clinical studies evaluating short-term plaque volume changes suggest that plaque regression is possible with intensive lipid lowering and intravascular imaging, these changes are small compared with control populations. In conclusion, we do not believe that plaque regression is the only therapeutic goal. We always believe that risk factors should be tightly controlled and treated early to prevent or minimize plaque progression and enhance its stability, regardless of whether plaque regression occurs. In addition, low-cost, low-risk circulating biomarkers have been independently associated with prognosis and may serve as an adjunct to identify patients more likely to benefit from lipid-lowering therapy.
